# Cholangiocarcinoma Metastasis to the Spine and Cranium

**DOI:** 10.31486/toj.18.0142

**Published:** 2020

**Authors:** Joshua A. Hanna, Mansour Mathkour, Edna E. Gouveia, Ryan Glynn, Adhira Divagaran, JonMark B. Lane, Cuong J. Bui, Olawale A. Sulaiman, Roger D. Smith

**Affiliations:** ^1^ Department of Neurosurgery, Ochsner Clinic Foundation, New Orleans, LA; ^2^ Department of Neurosurgery, Tulane Medical Center, New Orleans, LA; ^3^ The University of Queensland Faculty of Medicine, Ochsner Clinical School, New Orleans, LA

**Keywords:** *Chemotherapy*, *cholangiocarcinoma*, *cranium*, *neoplasm metastasis, radiotherapy*, *resection*, *spine*

## Abstract

**Background:** Cholangiocarcinoma (CCC), a rare tumor arising from the viscera, has a poor prognosis. Although CCC is prone to metastasis, spread to the cranium and spine is exceedingly rare. Treatment for metastatic disease is palliative, with total resection of the primary lesion the only cure. We describe a case of metastatic CCC to the spine and cranium treated with surgical resection.

**Case Report:** A 61-year-old male with a history of hepatitis C with liver transplant and incidental discovery of CCC presented with gradually increasing back pain. Physical examination revealed a palpable nontender mass in the parieto-occipital area. Computed tomography survey of the spine and head revealed mixed sclerotic and lytic lesions of the T9, T11, L2, and L5 vertebral bodies, a lytic lesion on the T6 vertebral body, and a 1.4-cm lesion in the right occipital calvarium. The patient underwent right occipital craniotomy for excisional biopsy of the calvarial mass with gross total resection and immunohistochemical confirmation of CCC. The patient was started on gemcitabine chemotherapy and radiation therapy for spinal metastases. Three months later, the patient died from metastatic disease complications.

**Conclusion:** To our knowledge, only 6 cases of cranial CCC have been reported, and only 2 reported mixed cranial/spinal involvement. We report a rare case of CCC metastasis to the spine and cranium that was treated with surgery, chemotherapy, and radiotherapy. CCC should be considered an exceedingly rare etiology with treatment options aimed solely at palliation. This case supplements the existing literature to inform medical and surgical decision-making.

## INTRODUCTION

Cholangiocarcinoma (CCC), a rare tumor that arises from the bile duct, accounts for only 3% of all gastrointestinal cancers^1^ and 15% of liver cancers.^2^ The prognosis for CCC is particularly poor when presenting with unresectable anatomy (5-year survival of approximately 12%).^3^ However, treatment with Whipple duodenopancreatectomy is possible in 67% of patients with extrahepatic CCC,^3,4^ and this treatment option increases the 5-year survival rate from 12% to 28%.^4^ In studies by He and Wu (2008) and by Ramírez-Merino et al (2013), radiotherapy and chemotherapy were trialed with questionable efficacy.^5,6^ CCC is known to metastasize most commonly to the liver, peritoneum, intraabdominal lymph nodes, and lungs.^7^ Bone metastases are relatively less common^8^ and most frequently occur in the distal skeleton.^9^ Concurrent metastasis to the spine and cranium is an exceedingly rare phenomenon with few reported cases to date.^3,10-15^

Metastasis of CCC to the cranium and spinal column is hypothesized to occur through 2 main pathways: (1) the osseous pathway via the craniospinal venous system (CSVS)^16,17^ and (2) hematogenous spread via the pulmonary vasculature to seed the axial skeleton and brain.^10,12^ The CSVS has 2 major divisions: (1) the vertebral venous system that includes the Batson plexus^16^ and (2) the intracranial veins that include the cavernous sinuses, the cortical veins, the dural sinuses, and the ophthalmic veins.^17^ The anatomy of the CSVS includes veins that exist without valves, allowing bidirectional flow^16,18^ with increased intraabdominal or intrathoracic pressure.^16-18^ Retrograde venous flow within the CSVS is currently the most supported theory for CCC metastasis to the cranium and spine as per Fujimoto et al, who demonstrated metastasis along the lines of the CSVS.^10^

Definitive diagnosis of CCC metastasis to the spine and cranium is reserved for pathology and immunohistochemistry, as radiologic imaging and clinical presentation are nonspecific. Despite poor specificity, positron emission tomography (PET) offers significant advantages as a method of detecting metastases,^10,12^ with methionine PET showing higher sensitivity than fluorodeoxyglucose PET.^10^ Magnetic resonance imaging (MRI) classically shows a heterointense honeycomb appearance on both T1- and T2-weighted imaging and may be used to assist in the planning of surgical resection.^12^ Common presenting symptoms are also largely nonspecific and may vary by location. Metastasis to the skull is typically asymptomatic but may cause severe disability with compression of the dural sinuses,^19^ disturbance of the cranial nerves,^20^ epidural hematoma,^21-23^ and expansion of the tumor secondary to intratumoral hemorrhage.^24^ Metastasis to the spine may present as neck pain,^11^ back pain,^15^ periscapular pain,^3^ or even complete neurologic syndrome.^25^ On cytologic examination, smears reveal a glandular arrangement of crowded clusters and sheets of malignant cells with large nuclei and prominent nucleoli.^11^ Immunohistochemistry of metastatic CCC cells shows strong immunopositivity for cytokeratin (CK)-7 and CK-19, AE1/AE3, CAM 5.2, MOC-31, and polyclonal carcinoembryonic antigen (pCEA).^11,26^ Equally important is the immunonegativity for thyroid transcription factor-1 (TTF-1), prostate-specific antigen (PSA), and CK-20 that helps to rule out metastasis from the lung, prostate, and colon, respectively.^11^

While documented evidence of CCC spine metastasis was previously limited, case series published between 2001 and 2018—largely from Thailand and South Korea—demonstrate hundreds of cases of CCC metastasis to the spine,^3,10-13,15,27-31^ yet only 6 cases of metastasis to the cranium have been reported,^10,12,14,32^ and only 2 of those cases coinvolved the spine and cranium.^10,12^ We present a case of combined CCC metastasis to the cranium and spine.

## CASE REPORT

A 61-year-old male presented with a 1-month history of increasing back pain that he associated with muscle spasm. The patient reported that the pain started gradually and had progressed to a 7/10 on the pain scale by the time he presented to the hospital. The patient's significant medical history included a liver transplant 9 months prior secondary to chronic hepatitis C. At the time of the patient's transplant, pathologic review of the explant was notable for a 0.6-cm pure hepatocellular carcinoma and a 0.7-cm mixed cholangiocarcinoma with hepatocellular carcinoma components, both confined to the right lobe of the liver. No adjuvant treatment was undertaken at the time of transplant, as both lesions were without lymphovascular invasion. Physical examination showed a palpable nontender mass in the parieto-occipital area but was otherwise unremarkable.

**Figure 1.**
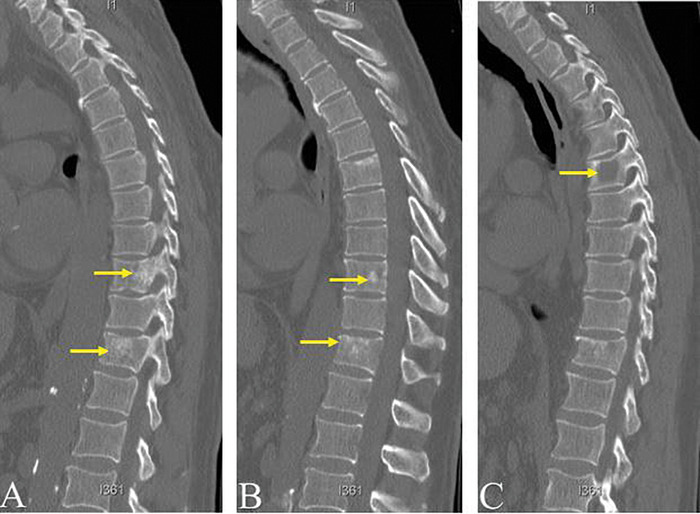
**Sagittal computed tomography sections without contrast of the thoracic spine demonstrate multiple mixed sclerotic/lytic lesions involving the T9 and T11 vertebral bodies (A and B), with a lytic lesion at T6 (C). Lumbar lesions are not shown.**

**Figure 2.**
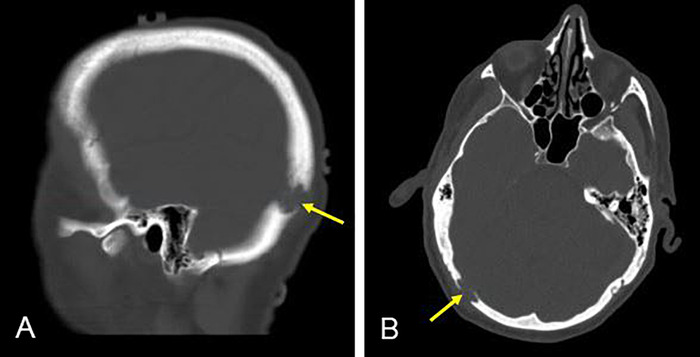
**Computed tomography head with contrast. Sagittal (left) and axial (right) views demonstrate a 1.4-cm focal bony erosive lesion in the right occipital calvarium with ill-defined margins. Deformity and defect extend through both tables of the calvarium.**

Computed tomography (CT) imaging of the spine revealed mixed sclerotic/lytic lesions involving T9 and T11 (Figures 1A and 1B) and L2 and L5; an additional lytic lesion was seen in the T6 vertebral body (Figure 1C). The T11 vertebral body appeared almost entirely replaced by sclerotic focus; the T9/T10 levels demonstrated extension into the central canal; and the lesion in the T6 vertebral body measured 1.7 cm. CT imaging of the head revealed a focal well-defined erosive lesion within the right occipital calvarium that measured approximately 1.4 cm and likely involved the inner and outer tables of the calvarium (Figure 2). Associated with this lesion was overlying soft tissue prominence concerning for extraosseous extension measuring 9 mm in thickness. No apparent local reaction in the brain was noted, and the cerebral parenchyma appeared otherwise unremarkable. MRI was not done at this time.

**Figure 3.**
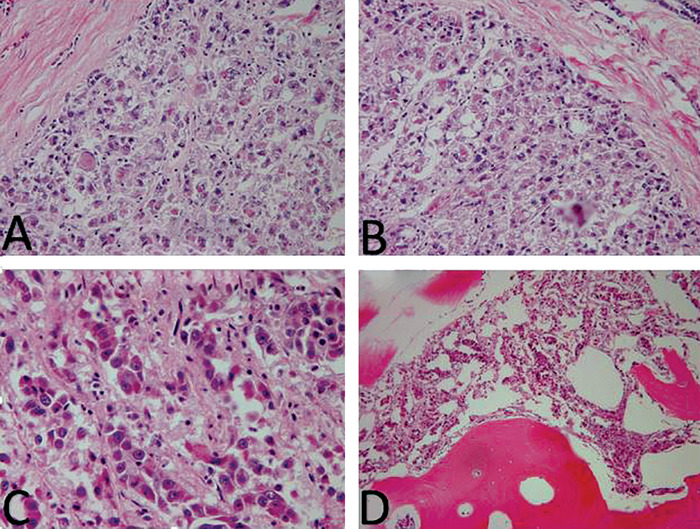
**Histopathology hematoxylin and eosin stains of the primary tumor initially found in the right lobe of the liver (A and B). Histopathology hematoxylin and eosin stains of the metastatic adenocarcinoma excised from the calvarium (C and D).**

The patient underwent a right occipital craniotomy with stealth navigation for excisional biopsy of the mass. Intraoperative visualization and resection of the tumor revealed a 1.4-cm red-brown mass that extended through both tables of the skull. The mass was adherent with but not attached to the dura and was easily scraped away. Special care was taken to clean and coagulate the involved dura. Resection was completed with bone edges free of tumor involvement, and the lesion was fully contained within the craniotomy excision. Pathology results demonstrated a metastatic adenocarcinoma virtually identical to the cholangiocarcinoma component seen in the patient's liver explant 9 months prior (Figure 3). Immunohistochemistry showed focal CK-7 positivity and negativity to hepatocyte antigen, CK-20, synaptophysin, and chromogranin. Postoperative imaging demonstrated complete resection of the cranial lesion. The patient progressed without complications and was discharged 4 days later.

The patient was started on cisplatin 25 mg/m^2^ and gemcitabine 1,000 mg/m^2^ infusions the same day as his craniotomy; he had a second and final chemotherapeutic treatment 1 week postoperatively. One month following his operation, the patient began a course of radiation to a dose of 30 Gy in fractions of 3 Gy that targeted the metastases to the thoracic spine and the L5 vertebral body. The patient continued follow-up with neurosurgery following his operation; however, he succumbed to his illness and died from complications of the metastatic disease 3 months postoperatively.

## DISCUSSION

Metastases from CCC display unique cytologic and immunohistochemical properties that aid in diagnosis^11,26^ but present with nonspecific symptoms^3,15,19-25^ and imaging.^10,12^ Metastasis to the spine and/or cranium is rare; however, a small number of cases with spinal and cranial metastasis have been documented in the literature in case reports (Table 1)^3,10-15^ and in retrospective clinical studies and review articles (Table 2).^27-32^

**Table 1.** Characteristics of Patients in Case Reports of Axial Cholangiocarcinoma Metastasis

	Age in				Metastasis	Surgical
Case	Years, Sex	Presenting Symptom	Interval	Location of Metastasis	to CNS	Resection
Current case	61, M	Back pain	9 months	Right occipital bone, T9-T11, L2, L5	No	Yes, cranial lesion
Faugeras et al,^3^ 2015	62, M	Pain in scapula	2 years	L2, C4	Yes	No
Purushothaman et al,^13^ 2015	40, F	Neck pain	Unknown	C6-T2	No	No
Fujimoto et al,^10^ 2013	56, F	Painful mass in right parietal region	3 years	Left orbit, left parietal bone, left temporal bone	No	No
Fujimoto et al,^10^ 2013	58, F	Pain and swelling in left orbit	2 months	Left orbit, right parietal bone	No	No
Fujimoto et al,^10^ 2013	65, M	Pain in right occipital region with right IX, X, XI, XII palsy	2 months	Right petrous bone, C1	No	No
Kidambi et al,^11^ 2011	82, M	Progressive neck pain	2 months	C3, C4, T3-T5	No	Unknown
Wojtas and Deinsberger,^14^ 2009	72, F	Headache	2 years	Cranium, unspecified	No	Yes
Miyamoto et al,^12^ 2007	67, F	Painful subcutaneous lesion in parietal region	2 years	Left occipital bone, T12	No	Yes, cranial lesion
Yeh et al,^15^ 2001	63, F	Low back pain	2 years	T12	No	Yes

CNS, central nervous system; F, female; M, male.

**Table 2.** Characteristics of Patients in Studies (Other Than Case Reports) of Axial Cholangiocarcinoma Metastasis

	Number of	Median	Most Common	Most Common	Metastasis
Study	Patients	Age, Years	Presenting Symptoms	Location of Metastasis	to CNS
Sangsin et al,^27^ 2018	182	57	Jaundice, hepatomegaly, palpable gallbladder and/or liver mass	Multilevel spine	No
Dowsiriroj et al,^28^ 2017	55	57	Neurologic deficit (Frankel scale grade C or lower)	Thoracic spine	No
Turel et al,^31^ 2017	2	57.5	Inability to ambulate	L2/S1	No
Goodwin et al,^29^ 2016	16	55.5	Pain	Multilevel spine	No
Singh et al,^32^ 2015	4	61.5	Pain	Cranium	No
Paholpak et al,^30^ 2012	15	57.5	N/A	Thoracic spine	No

CNS, central nervous system; N/A, not available.

Yeh et al described the first incidence of CCC metastasis to the spine in 2001,^15^ followed by the first reported case of skull metastasis by Miyamoto et al in 2007.^12^ Including our own patient, there are currently 9 known cases of CCC metastasis to the spine in the United States,^3,10-13,15,31^ with hundreds more reported in Eastern Asia, and 7 cases of metastasis to the cranium^10,12,14,32^ with 3 combined cases of spine/cranium metastasis including our own.^10,12^ In these cases, the average patient age was 60.1 years with a range of 38 to 82 years and a female:male ratio of approximately 1:1 (Tables 1 and 2).

Although it is tempting to believe that spinal/cranial metastasis from CCC is a novel presentation of an existing tumor entity, we posit that the historic absence of immunohistochemistry to aid in past diagnoses may have obscured previous cases in the literature. Other sources argue that the increasing incidence of these less common metastatic sites may be attributable to an increase in overall survival of patients secondary to the advent of multimodal therapies.^3^ This increase in incidence is supported by studies from 2010 and 2013^6,33^ that correlated prolonged overall survival and progression-free survival with modern management strategies, particularly the use of cisplatin and gemcitabine in palliative situations. This theory is supported by the fact that 6 patients, including our own, had prolonged latency periods between the original presentation of CCC and metastasis to the spine and/or cranium. However, 4 patients presented with metastasis concomitant with the discovery of their primary neoplasm.^10-15^

Pending definitive diagnosis with cytology and immunohistochemistry, the 4 treatments for metastatic CCC are surgery, radiation, chemotherapy, and hormonal therapy.^10^ Of these, surgical excision is most commonly employed for symptomatic lesions, provided the lesion can be removed with little risk of morbidity or mortality.^19,34,35^ The goal of surgery in metastatic disease is to offer local control and symptomatic relief, with no expected change in overall prognosis.^25,35^ When lesions are too extensive or too difficult to resect, radiotherapy may be applied.^10^ Radiotherapy with or without chemotherapy was found to provide pain relief in up to 90% of patients with symptoms of short duration.^36^ Chemotherapy and hormonal therapy are considered to be the least effective treatments overall^3,5,6,10^; however, in studies from 2011 and 2017, the use of gemcitabine and cisplatin together demonstrated positive effects on systemic lesions and potential clinical improvement.^28,37^

As mentioned previously, the use of Whipple duodenopancreatectomy in extrahepatic CCC has increased the overall 5-year survival rate from 12% to 28% in local disease.^4^ However, improvements have also been found in metastatic disease. In CCC with metastasis, median survival is currently 7 to 12 months, with a 2013 study from Ramírez-Merino et al showing even longer overall survival and progression-free survival with newer treatment regimens.^6^ Still, the exact impact of cranial and/or spinal metastases on disease progression, morbidity, and mortality has yet to be established. The authors anticipate a negative prognostic effect secondary to the neurologic sequelae described in this report.

## CONCLUSION

CCC is a rare visceral tumor, not classically associated with spread to the spine and/or cranium. CCC metastasis is indistinguishable from more common primary tumor types by clinical or radiologic findings, making preoperative diagnosis impossible without the assistance of pathologic study. CCC metastasis to the cranium or spine has indeterminate impact on the disease course and prognosis, although our own experience demonstrates a poor prognostic outcome. Upon diagnosis, treatment of metastatic sites is considered solely palliative but may be combined with curative measures. This case supplements the existing literature to inform medical and surgical decision-making.

## Notes

This article meets the Accreditation Council for Graduate Medical Education and the American Board of Medical Specialties Maintenance of Certification competencies for Patient Care and Medical Knowledge.

## References

[1] Khan S, Thomas HC, Davidson BR, Taylor-Robinson SD Cholangiocarcinoma. Lancet. 2005 Oct 8;336(9493):1303-1314. doi: 10.1016/S0140-6736(05)67530-7. 16214602

[2] Chen MF Peripheral cholangiocarcinoma (cholangiocellular carcinoma): clinical features, diagnosis and treatment. J Gastroenterol Hepatol. 1999 12;14(12):1144-1149. 10634149 10.1046/j.1440-1746.1999.01983.x

[3] Faugeras L, Cantineau G, Daisne J, Gustin T, D'hondt L Intramedullary spinal cord metastasis of cholangiocarcinoma: a case report. BMC Res Notes. 2015 Feb 14;8:41. doi: 10.1186/s13104-015-0998-y. 25889352 PMC4340695

[4] Tompkins RK, Thomas D, Wile A, Longmire WP Jr. Prognostic factors in bile duct carcinoma: analysis of 96 cases. Ann Surg. 1981 10;194(4):447-457. 7283506 10.1097/00000658-198110000-00008 PMC1345321

[5] He XR, Wu XP Difference in biological characteristics and sensitivity to chemotherapy and radiotherapy between intrahepatic and extrahepatic cholangiocarcinoma cells in vitro. Chin Med Sci J. 2008 Mar;23(1):54-59. 18437912 10.1016/s1001-9294(09)60011-0

[6] Ramírez-Merino N, Aix SP, Cortés-Funes H Chemotherapy for cholangiocarcinoma: an update. World J Gastrointest Oncol. 2013 Jul 15;5(7):171-176. doi: 10.4251/wjgo.v5.i7.171. 23919111 PMC3731530

[7] Wakahara T, Tsukamoto T, Kitamura S, Metastatic colon cancer from intrahepatic cholangiocarcinoma. J Hepatobiliary Pancreat Surg. 2005;12(5):415-418. doi: 10.1007/s00534-005-0991-2. 16258812

[8] Habermehl D, Haase K, Rieken S, Debus J, Combs SE Defining the role of palliative radiotherapy in bone metastasis from primary liver cancer: an analysis of survival and treatment efficacy. Tumori. 2011 Sep-Oct;97(5):609-613. 22158492 10.1177/030089161109700512

[9] Lahrach K, Chbani B, Amar F, Bennani A, Marzouki A, Boutayeb F Humerus pathological fracture revealing biliary carcinoma. Orthop Traumatol Surg Res. 2010 12;96(8):910-912. doi: 10.1016/j.otsr.2010.05.011. 21056026

[10] Fujimoto K, Kuroda J, Makino K, Hasegawa Y, Kuratsu J Skull metastasis from intrahepatic cholangiocarcinoma: report of 3 cases and review of the literature. Neurol Med Chir (Tokyo). 2013;53(10):717-721. 24077267 10.2176/nmc.cr2012-0237 PMC4508741

[11] Kidambi T, Mahajan A, DiBardino D Cholangiocarcinoma presenting as metastases to the cervical spine. Am J Med. 2011 5;124(5):e1-e2. doi: 10.1016/j.amjmed.2010.12.011. 21531219

[12] Miyamoto J, Tatsuzawa K, Sasajima H, Mineura K Metastatic skull tumor from cholangiocarcinoma. Case report. Neurol Med Chir (Tokyo). 2007 Mar;47(3):132-135. 17384497 10.2176/nmc.47.132

[13] Purushothaman B, Sewell MD, Williams R Cholangiocarcinoma presenting as cervical spine metastasis. Spine J. 2015 Jul 1;15(7):1699. doi: 10.1016/j.spinee.2015.03.034. 25819587

[14] Wojtas K, Deinsberger W Giant cholangiocarcinoma skull metastasis with intracranial and extracranial location – an unusual case report. Abstract presented at: American Association of Neurological Surgeons 77th Annual Meeting; August 30, 2009; San Diego, CA www.aans.org/Annual-Scientific-Meeting/Abstract-Center/Abstract-Details?page=1&id=58979&SearchTerm=Giant%20cholangiocarcinoma. Accessed January 31, 2020.

[15] Yeh CN, Chen MF, Chen TC, Tseng JH Peripheral cholangiocarcinoma with thoracic spine metastasis: a successful surgically treated case. Int Surg. 2001 Oct-Dec;86(4):225-228. 12056466

[16] Batson OV The function of the vertebral veins and their role in the spread of metastases. Ann Surg. 1940 7;112(1):138-149. 17857618 10.1097/00000658-194007000-00016 PMC1387927

[17] Tobinick E, Vega CP The cerebrospinal venous system: anatomy, physiology, and clinical implications. MedGenMed. 2006 Feb 22;8(1):53. 16915183

[18] Coman DR, DeLong RP The role of the vertebral venous system in the metastasis of cancer to the spinal column: experiments with tumor-cell suspensions in rats and rabbits. Cancer. 1951 5;4(3):610-618. 14839615 10.1002/1097-0142(195105)4:3<610::aid-cncr2820040312>3.0.co;2-q

[19] Michael CB, Gokaslan ZL, DeMonte F, McCutcheon IE, Sawaya R, Lang FF Surgical resection of calvarial metastases overlying dural sinuses. Neurosurgery. 2001 4;48(4):745-754; discussion 754-755. 11322434 10.1097/00006123-200104000-00009

[20] Wakisaka S, Tashiro M, Nakano S, Kita T, Kisanuki H, Kinoshita K Intracranial and orbital metastasis of hepatocellular carcinoma: report of two cases. Neurosurgery. 1990 5;26(5):863-866. 2162018 10.1097/00006123-199005000-00021

[21] Hayashi K, Matsuo T, Kurihara M, Daikoku M, Kitange G, Shibata S Skull metastasis of hepatocellular carcinoma associated with acute epidural hematoma: a case report. Surg Neurol. 2000 4;53(4):379-382. 10825524 10.1016/s0090-3019(00)00208-1

[22] Nakagawa Y, Yoshino E, Suzuki K, Tatebe A, Andachi H Spontaneous epidural hematoma from a hepatocellular carcinoma metastasis to the skull–case report. Neurol Med Chir (Tokyo). 1992 5;32(5):300-302. 1378949 10.2176/nmc.32.300

[23] Nakao N, Kubo K, Moriwaki H Cranial metastasis of hepatocellular carcinoma associated with chronic epidural hematoma—case report. Neurol Med Chir (Tokyo). 1992 2;32(2):100-103. 1376859 10.2176/nmc.32.100

[24] Shibukawa M, Inagawa T, Katoh Y, Tokuda Y, Ohbayashi N, Yoshioka Y A case of cranial metastasis of hepatocellular carcinoma [in Japanese]. No To Shinkei. 1995 11;47(11):1087-1091. 7495615

[25] Constans JP, de Divitiis E, Donzelli R, Spaziante R, Meder JF, Haye C Spinal metastases with neurological manifestations. Review of 600 cases. J Neurosurg. 1983 7;59(1):111-118. doi: 10.3171/jns.1983.59.1.0111. 6864265

[26] Lau SK, Prakash S, Geller SA, Alsabeh R Comparative immunohistochemical profile of hepatocellular carcinoma, cholangiocarcinoma, and metastatic adenocarcinoma. Hum Pathol. 2002 12;33(12):1175-1181. doi: 10.1053/hupa.2002.130104. 12514785

[27] Sangsin A, Saiudom D, Pongmanee S, Saengsin J, Leerapun T, Murakami H Natural history and prognostic factors of cholangiocarcinoma with spinal metastasis: a 10-year single center study. Clin Spine Surg. 2018 4;31(3):E160-E165. doi: 10.1097/BSD.0000000000000625. 29596214 PMC5895169

[28] Dowsiriroj P, Paholpak P, Sirichativapee W, Cholangiocarcinoma with spinal metastasis: single center survival analysis. J Clin Neurosci. 2017 4;38:43-48. doi: 10.1016/j.jocn.2016.12.048. 28108084

[29] Goodwin CR, Abu-Bonsrah N, Boone C, Non-hepatocellular carcinoma spinal metastases. J Clin Neurosci. 2016 5;27:22-27. doi: 10.1016/j.jocn.2015.11.003. 26778049

[30] Paholpak P, Sirichativapee W, Wisanuyotin T, Kosuwon W, Jeeravipoolvarn P Prevalence of known and unknown primary tumor sites in spinal metastasis patients. Open Orthop J. 2012;6:440-444. doi: 10.2174/1874325001206010440. 23115604 PMC3480984

[31] Turel MK, Kerolus MG, O’Toole JE Minimally invasive “separation surgery” plus adjuvant stereotactic radiotherapy in the management of spinal epidural metastases. J Craniovertebr Junction Spine. 2017 Apr-Jun;8(2):119-126. doi: 10.4103/jcvjs.JCVJS_13_17. 28694595 PMC5490345

[32] Singh M, Ricci JA, Talbot SG, Chiocca EA, Dunn IF, Caterson EJ Reconstruction of rare skull metastases using free latissimus dorsi flap and the role of preoperative embolization in hypervascular skull tumors. J Craniofac Surg. 2015 11;26(8):2289-2292. doi: 10.1097/SCS.0000000000002218. 26501975

[33] Valle J, Wasan H, Palmer DH, et al; ABC-02 Trial Investigators. Cisplatin plus gemcitabine versus gemcitabine for biliary tract cancer. N Engl J Med. 2010 Apr 8;362(14):1273-1281. doi: 10.1056/NEJMoa0908721. 20375404

[34] Laigle-Donadey F, Taillibert S, Martin-Duverneuil N, Hildebrand J, Delattre JY Skull-base metastases. J Neurooncol. 2005 10;75(1):63-69. doi: 10.1007/s11060-004-8099-0. 16215817

[35] Stark AM, Eichmann T, Mehdorn HM Skull metastases: clinical features, differential diagnosis, and review of the literature. Surg Neurol. 2003 9;60(3):219-225; discussion 225-226. 12922038 10.1016/s0090-3019(03)00269-6

[36] Vikram B, Chu FC Radiation therapy for metastases to the base of the skull. Radiology. 1979 2;130(2):465-468. doi: 10.1148/130.2.465. 104361

[37] Mitsuya K, Nakasu Y, Horiguchi S, Metastatic skull tumors: MRI features and a new conventional classification. J Neurooncol. 2011 8;104(1):239-245. doi: 10.1007/s11060-010-0465-5. 21110218 PMC3151370

